# IL-6-Inducing Peptide Prediction Based on 3D Structure and Graph Neural Network

**DOI:** 10.3390/biom15010099

**Published:** 2025-01-10

**Authors:** Ruifen Cao, Qiangsheng Li, Pijing Wei, Yun Ding, Yannan Bin, Chunhou Zheng

**Affiliations:** 1 Information Materials and Intelligent Sensing Laboratory of Anhui Province, School of Computer Science and Technology, Anhui University, Hefei 230601, China; rfcao@ahu.edu.cn (R.C.); e22301211@stu.ahu.edu.cn (Q.L.); 2 Institutes of Physical Science and Information Technology, Anhui University, Hefei 230601, China; weipj@ahu.edu.cn; 3 School of Artificial Intelligence, Anhui University, Hefei 230601, China; yunding@ahu.edu.cn

**Keywords:** interleukin-6, IL-6-inducing peptides, graph attention network, graph convolutional network, ESM-1b

## Abstract

Interleukin-6 (IL-6) is a potent glycoprotein that plays a crucial role in regulating innate and adaptive immunity, as well as metabolism. The expression and release of IL-6 are closely correlated with the severity of various diseases. IL-6-inducing peptides are critical for the development of immunotherapy and diagnostic biomarkers for some diseases. Most existing methods for predicting IL-6-induced peptides use traditional machine learning methods, whose feature selection is based on prior knowledge. In addition, none of these methods take into account the three-dimensional (3D) structure of peptides, which is essential for their functional properties. In this study, we propose a novel IL-6-inducing peptide prediction method called DGIL-6, which integrates 3D structural information with graph neural networks. DGIL-6 represents a peptide sequence as a graph, where each amino acid is treated as a node, and the adjacency matrix, representing the relationships between nodes, is derived from the predicted residue contact graph of the peptide sequence. In addition to commonly used amino acid representations, such as one-hot encoding and position encoding, the pre-trained model ESM-1b is employed to extract amino acid features as node features. In order to simultaneously consider node weights and information updates, a dual-channel method combining Graph Attention Network (GAT) and Graph Convolutional Network (GCN) is adopted. Finally, the extracted features from both channels are merged for the classification of IL-6-inducing peptides. A series of experiments including cross-validation, independent testing, ablation studies, and visualizations demonstrate the effectiveness of the DGIL-6 method.

## 1. Introduction

Interleukin-6 (IL-6), a genetically encoded 184-amino acid quadruple helical protein, is a key pro-inflammatory cytokine [1,2]. It is also commonly referred to as interferon- β2 (IFN- β2), B-cell-stimulating factor-2, and hybridoma/plasmacytoma growth factor. As a pleiotropic cytokine, IL-6 is produced by various cell types, including T cells, fibroblasts, monocytes, endothelial cells, macrophages, and dendritic cells [3,4,5]. This cytokine plays a critical role in adaptive and innate immune responses, hematopoiesis, acute phase response, inflammatory diseases, and organ development [6]. Furthermore, cytokine burst or cytokine release syndrome is closely related to the progression of COVID-19. In patients with COVID-19, significantly elevated levels of IL-6 and other pro-inflammatory cytokines, such as IL-7, IL-8, and IL-12, are strongly related to disease progression [7]. It should be noted that the *SARS-CoV-2* virus can selectively induce IL-6 overproduction, leading to lymphocyte depletion [8]. IL-6-inducing peptides play a crucial role in the regulation of IL-6 expression and activity, and their interactions significantly impact immune balance and disease progression [9]. Therefore, accurate identification of IL-6-inducing peptides is essential to deepen our understanding of IL-6-mediated mechanisms and provide valuable information for the development of diagnostic tools and immunotherapy strategies.

Several machine learning approaches have been developed in recent years to predict IL-6-inducing peptides. Dhall et al. introduced IL-6Pred, which uses 15 peptide feature descriptors to map sequences to feature vectors, and then input them into a random forest model for prediction [10]. To further improve predictive performance, Charoenkwan et al. proposed StackIL6, a stacked ensemble model that combines three primary peptide features with five well-known traditional machine learning models [11]. By fine-tuning the hyperparameters of individual classifiers, StackIL6 significantly enhances both recognition accuracy and generalization capabilities. However, machine learning-based methods heavily rely on extensive human driven feature engineering and domain expertise. Deep learning approaches have also advanced the prediction of IL-6-inducing peptides. MVIL6, a deep learning-based predictor, leverages peptide sequence information by combining the Transformer architecture with the pre-trained MG-BERT model, effectively extracting feature representations and improving recognition performance [12]. Despite these promising advancements, existing studies largely overlook the structural features of IL-6-inducing peptides, which are crucial for their functional roles [13,14,15,16].

To address the limitations of existing methods, we propose a novel tool for IL-6-inducing peptide prediction, named DGIL-6, which leverages graph neural networks and 3D structural information. DGIL-6 models a peptide sequence as a graph, where each node represents an amino acid, and edges are defined by the adjacency matrix based on the predicted 3D peptide structure. In addition to conventional amino acid feature extraction methods, such as one-hot encoding and position encoding, DGIL-6 employs a pre-trained model, ESM-1b [17], to extract amino acid features as node attributes. After obtaining the graph representation of a peptide, a dual-channel graph network architecture is utilized to extract features. Specifically, the graph attention network (GAT) [18] is applied to update node features, while the graph convolutional network (GCN) [19] focuses on updating node weights. The features obtained from both channels are subsequently fused to classify IL-6-inducing peptides effectively.

## 2. Materials and Methods

### 2.1. Data Collection

**Figure 1:**
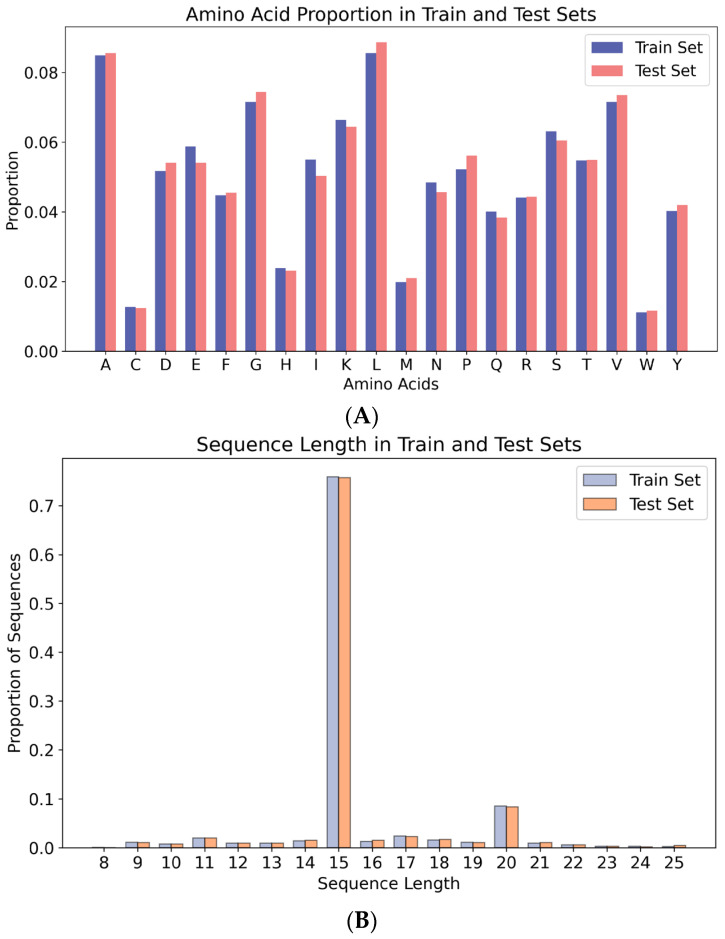
(**A**) Amino Acid proportion in training and testing sets. (**B**) Sequence-length proportion distribution in training and testing sets.

To effectively compare our method with previous studies, we used the same baseline dataset as Dhall et al. (2020) [10]. This dataset was selected due to its rigorous validation and widespread use in research on IL-6-inducing peptide recognition. DHall et al. extracted 583 experimentally validated IL-6-inducing peptides from the Immune Epitope Database (IEDB) [20]. Identical peptides and those longer than 25 amino acids were excluded, as most IL-6-inducing peptides are shorter than 25 amino acids. After filtering, 365 IL-6-inducing peptides (referred to as positive data) were retained; these had been tested in human or mouse hosts. For the negative dataset, Dhall et al. extracted experimentally validated peptides from IEDB that induced other cytokines, such as IL-1 α, IL-1 β, TNF α, IL-8, IL-12, IL-17, and IL-18, but not IL-6. These peptides, known as non-IL-6-inducing peptides, were used as negative samples, since they do not induce IL-6. The negative dataset consists of sequences have been tested either in human or mouse hosts. As with the positive peptides, identical peptides and those longer than 25 amino acids were excluded. Ultimately, 2991 non-IL-6-inducing peptides were retained. This dataset consists of 365 IL-6-inducing peptides and 2991 non-IL-6-inducing peptides, serving as the benchmark for studies on IL-6-inducing peptide prediction tools. The imbalance between positive and negative samples may affect the model’s performance with regard to the minority class. Moreover, peptides with IL-6 inducing activity are generally much smaller than those without IL-6 induction in real-world datasets. To address this issue, we employed balancing techniques during training, such as weighting the positive and negative samples, to improve the model’s ability to recognize the minority class (IL-6-inducing peptides). Several studies have previously used an 80:20 split ratio for training and testing datasets [21,22,23,24]. Following this standard protocol, 80% of the data were used for training, and 20% were reserved for testing. The training set contained 292 IL-6-inducing peptides and 2393 non-IL-6-inducing peptides, while the test set comprised 73 IL-6-inducing peptides and 598 non-IL-6-inducing peptides. In order to better understand the distribution of samples in the training and testing sets, we conducted amino acid composition analysis (Figure 1A) and amino acid length analysis (Figure 1B) on them. The results showed comparable amino acid compositions between the two sets, with no significant biases in residue frequencies. Similarly, the sequence length distributions were consistent, confirming the fairness of the dataset split. The specific details of the dataset are provided in Table 1.

### 2.2. Overview of DGIL-6

**Figure 2:**
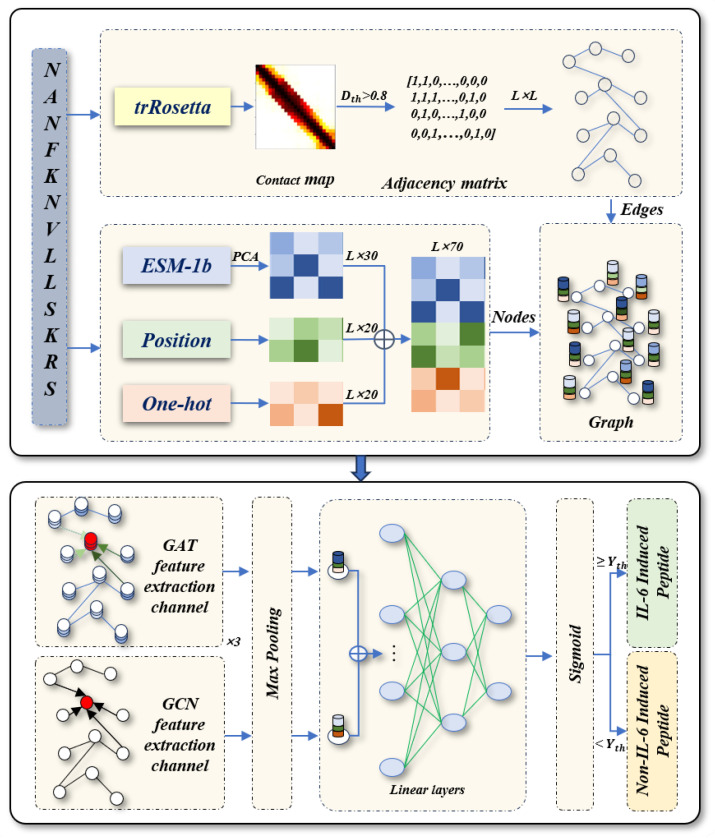
The network architecture of DGIL-6.

The framework of the DGIL-6 method is depicted in Figure 2 and consists of three main components: graph representation, a dual-channel feature extraction module, and a classification module. Graph representation: Amino acids in the peptide sequence are encoded as node features using one-hot encoding, position encoding, and features extracted by the pre-trained ESM-1b model. Structural features are derived from the peptide’s structural information, and an adjacency matrix is constructed based on the residue contact map. Together, these elements form a graph representation of the peptide sequence, where nodes represent amino acids, and edges reflect their structural relationships. Dual-channel feature extraction module: The graph representation is input into a dual-channel feature extraction module. One channel utilizes a multi-head Graph Attention Network (GAT) to update node features, while the other channel employs a Graph Convolutional Network (GCN) to update node weights. This dual-channel design captures complementary information, enabling more robust feature extraction. Classification module: The features extracted from the dual-channel module are fused and passed to the classification module, which predicts whether a peptide induces IL-6. Details of each component are provided in the subsequent sections.

### 2.3. Graph Representation

To investigate the role of peptide structural information for identifying IL-6-inducing peptides, we represent each peptide as a graph G=(V,E), where *V* represents the sets of nodes (amino acids) and *E* denotes the set of edges (amino acid contacts), respectively. Representing peptides as graphs enables the model to capture the structural relationships between amino acids in peptide sequences. Each amino acid is treated as a node in the graph, and the edges between nodes represent the interactions or spatial relationships between amino acids. This graph-based representation incorporates three-dimensional structural information, providing a more comprehensive understanding of peptide features. The features of each graph node are generated by concatenating one-hot encoding, position encoding, and ESM-1b extracting features. For a graph containing *N* nodes, the node feature is $x_{1},x_{2},...,x_{N}$, $x_{i}\in R^{D}$, i∈(1,N) and it *D* is the dimensions of the features. The spatial relationships between nodes are encoded in an adjacency matrix *A*, where $A_{ij}=1$ if there is an edge between nodes *i* and *j*; otherwise, $A_{ij}=0$. The construction of node features and adjacency matrices used in this study will be described in detail in the following sections.

#### 2.3.1. Node Feature Embedding

##### Amino Acid Composition Representation Based on One-Hot Encoding

One-hot encoding is employed to represent the 20 standard amino acids as numerical vectors. In this encoding scheme, a value of one is assigned to the position corresponding to a given amino acid, while all other positions in the vector are set to zero. For instance, the encoding of the amino acid “A” is represented as ${\left[1,0,0,0,\ldots ,0\right]}_{20}$, which means each amino acid is represented by a 20-dimensional vector. This encoding method maps discrete feature values to points in Euclidean space, where each unique value corresponds to a specific position. By placing discrete features in Euclidean space, one-hot encoding facilitates more meaningful distance calculations.

##### Residue Position Encoding

Position encoding in many applications is considered an effective descriptor [25] calculated as follows:

(1) $$P\left(\mathrm{pos},2i\right)=\sin\left(\frac{\mathrm{pos}}{b^{2i/d}}\right)$$

(2) $$P\left(\mathrm{pos},2i+1\right)=\cos\left(\frac{\mathrm{pos}}{b^{2i/d}}\right)$$

Let pos (where 0≤pos<L) denote the position of the amino acid in a length of *L*, and let 2i and 2i+1 (where 0≤i<10) represent the individual positions in the corresponding position vector. The values at even positions are calculated using the sine function, while those at odd positions are calculated using the cosine function. Alternating between sine and cosine functions enhances the model’s robustness and performance. Here, *b* and *d* are constants, and are set to 1000 and 20, respectively. Each amino acid is represented by a 20-dimensional vector.

##### ESM-1b Feature Encoding

To comprehensively capture the features of each amino acid, this study utilizes the pre-trained ESM-1b model. ESM-1b is a high-capacity transformer model, optimized through hyperparameter tuning, that takes protein sequences as input. The embeddings generated by ESM-1b not only encode direct information about protein sequences but also implicitly capture deeper biological insights, including structural, functional, and evolutionary knowledge. The pre-trained ESM-1b model can uncover information that is absent in one-hot encoding or residue position encoding. The features from these encoding methods are combined to represent each amino acid [17]. The feature matrix derived from ESM-1b has a size of L×1280 (where *L* is the length of a peptide sequence), while both one-hot encoding and position encoding feature matrices have a size of L×20. Due to the substantial dimensionality difference between these feature types, directly concatenating the ESM-1b features with the other two could result in the ESM-1b features dominating, leading to poor performance. Thus, it is necessary to reduce the dimensionality of the ESM-1b features to match the scale of the other feature sets. Principal Component Analysis (PCA) [26] was used for dimensionality reduction, with the optimal dimension selected through experiments. Further details are provided in Section 3.3.

#### 2.3.2. Adjacency Matrix Construction

The adjacency matrix represents the relationships between nodes in the graph, which, in this study, corresponds to the relationship between two amino acids. To more accurately capture this relationship, we construct the adjacency matrix using predicted contact maps from trRosetta [27]. First, we query the sequence against the uniclust30_2018_08 database using the HHblits tool [28], generating a multiple sequence alignment (MSA). The MSA is then input into the trRosetta tool to predict the distributions of distances and orientations between residue pairs. The distance information represents the spatial relationship between two atoms in the contact map. Distances ranging from 2 Å to 20 Å are divided into 36 segments, with each representing a 0.5 Å increment, with an additional segment for residues that do not come into contact. TrRosetta predicts the contact probability for residues within each segment. In previous work, the segment with the highest probability among all equally spaced segments was used to represent residue distance; when the distance between two atoms in the contact map is less than or equal to a predefined threshold, it indicates that the residues are in contact [27]. However, this approach is not optimal. Therefore, the method proposed in this study sums the probabilities of all segments corresponding to distances between 2 and 8 Å to represent the contact probability between residues, and uses this to calculate the adjacency matrix $A_{ij}$ of the peptide. The calculation can be expressed as follows:

(3) $$C_{ij}=\sum_{n=1}^{13}dist_{ij}$$

**Figure 3:**
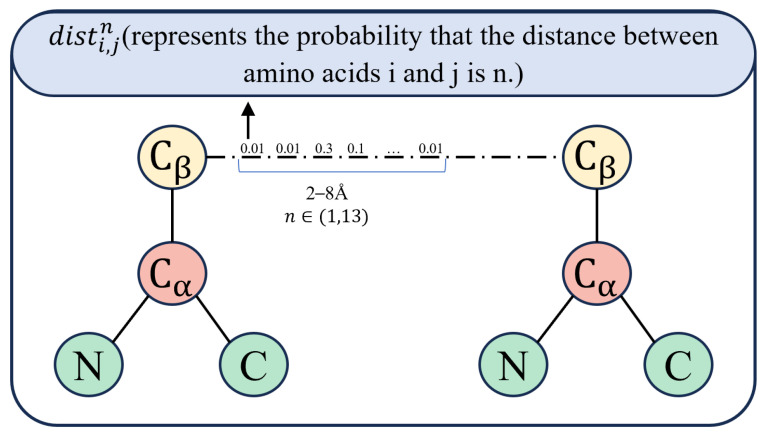
Calculation method for amino acid contact probability.

As shown in Figure 3, $dist_{ij}$ represents the probability distribution $C_{ij}$, where *n* = 1 and *n* = 13 correspond to distances of 2 Å and 8 Å, respectively. Residue pairs with contact probabilities $C_{ij}$ between residues greater than $C_{ij}$ = 0.8 are added to the graph as edge information:

(4) $$A_{ij}=\left\{\begin{array}{cc} 1, & \mathrm{if}\;C_{ij}\geq D_{\mathrm{th}}\;\mathrm{or}\;i=j,\\ 0, & \mathrm{otherwise}. \end{array}\right.$$

Here, $C_{ij}$ represents the contact probability between amino acids *i* and *j*, and $D_{th}$ denotes the contact threshold used in this study. As discussed in Section 3.4.3, setting this threshold to 0.8 yields the best performance, likely because it helps filter out improbable spatial interactions, thereby improving the precision of protein structure predictions.

### 2.4. Dual-Channel Feature Extraction

#### 2.4.1. The GAT Feature Extraction Channel

In this study, we use a multi-head GAT to model the weights of adjacent nodes and learn the hidden representations of nodes within the graph. The GAT utilizes a masked self-attention mechanism to assign weights to neighboring nodes, reflecting the importance of their connections. Specifically, GAT is employed to capture local correlations between residues and their neighbors. Due to errors in polypeptide structure prediction, the actual contact probabilities may deviate from the predicted ones. GAT can adaptively assign lower weights to these noisy data, thereby minimizing their negative impact on structural prediction outcomes. The process of using multiple iterations of the multi-head attention layer to calculate node features for the subsequent layer is as follows:

(5) $$H_{i}^{\prime }={\left|\right|}_{k=1}^{K}\sigma \left(\sum_{v_{j}\in N\left(v_{i}\right)}\alpha_{ij}^{\left(k\right)}W^{\left(k\right)}H_{j}\right)$$

where $H_{i}^{\prime }$ represents the feature representation of node *i*, *K* denotes the number of heads subjected to multi-head attention, σ the symbol stands for the activation function, $N(v_{i})$ represents the neighboring nodes of node *i*, and $W^{\left(k\right)}$ denotes the projection transformation matrix of the node features of the head *i*. $\alpha_{ij}^{\left(k\right)}$ is computed as follows:

(6) $$\alpha_{ij}^{\left(k\right)}=\frac{exp\left(g\left(a^{(k)T}\left[W^{\left(k\right)}H_{i}\left|\right|W^{\left(k\right)}H_{j}\right]\right)\right)}{\sum_{u\in N_{\left(i\right)}}exp\left(g(a^{(k)T}[W^{\left(k\right)}H_{i}\left|\right|W^{\left(k\right)}H_{u}]\right))}$$

where a(·) denotes a vector of learnable parameters, g(·) denotes a ReLU [29] activation function, and || denotes a cascade.

In this study, the graph information is input into a three-layer GAT with eight attention heads to obtain node weights. Layer normalization is applied to reduce covariate shift between layers, enhancing both training efficiency and the generalization of the model. ReLU is used as the activation function for the GAT layers. Following this, a global max pooling layer is introduced to select the global maximum after the GAT layers, thereby capturing the most representative information from the entire graph. In addition, a dropout layer [30] is employed to randomly discard neurons, further improving the generalization of the model.

#### 2.4.2. The GCN Feature Extraction Channel

The GCN fully aggregates information from adjacent nodes along with its own node information, enabling the node features to be updated. Therefore, the graph constructed from peptide sequences is input into the GCN feature-learning module developed in this study, allowing the model to focus on updating node features and learning effective representations. The GCN takes two primary inputs: the node feature matrix *X* and the adjacency matrix *A*. For a graph with *N* nodes, each node has *D*-dimensional features, which can be organized into an *N* × *D* matrix *X*. Additionally, the relational connections between nodes are represented by an *N* × *N* adjacency matrix *A*.

The weight update process in the GCN can be described by the following formula:

(7) $$H^{\prime }=\sigma \left({\tilde{D}}^{-1/2}\tilde{A}{\tilde{D}}^{-1/2}HW\right)$$

Let $H\in R^{N\times D}$, where *N* represents the number of nodes in the graph, and *D* is the dimensionality of the vector used to represent each node. The input layer consists of *H* and the adjacency matrix $\tilde{A}$, which includes self-connections. $\tilde{D}$ denotes the degree matrix of $\tilde{A}$, and *W* represents the trainable weight parameters. The symbol σ refers to the ReLU activation function. By applying this formula, the output graph representation $H^{\prime }$ is obtained. After the graph convolution, a global Max-pooling layer is used to select the global maximum, which captures the most informative feature of the entire graph.

### 2.5. Classification Module

The classification module uses the fused features obtained from the dual-channel feature extraction module, employing four fully connected layers and a sigmoid function to achieve the final classification of IL-6-inducing peptides. The specific implementation process is as follows:

(8) $$x_{0}=concatenate\left(maxpooling\left(H_{i}^{\prime }\right),maxpooling\left(H^{\prime }\right)\right)$$

(9) $$x_{i}=W_{i}x_{i-1}+b_{i}$$

(10) $$y_{i}=\frac{1}{1+e^{-x_{i}}}$$

Let $W_{i}$ denote the weight matrix of the *i*th linear layer, while $b_{i}$ represents the bias of the *i*th linear layer. Dropout and ReLU activation functions are applied after each linear layer to process the features. The model’s output, $y_{i}$, is obtained through the sigmoid activation function. Finally, a threshold $Y_{th}=0.35$ is set to determine whether the input sequence is an IL-6-inducing peptide.

The BCEWithLogitsLoss function, which combines a sigmoid function and a binary cross-entropy loss, is used during training. The formula of BCEWithLogitsLoss can be expressed as follows:

(11) $$\mathrm{Loss}=\frac{1}{N}\sum_{i=1}^{N}\left(y_{i}\cdot \log\left(\sigma (p_{i})\right)+\left(1-y_{i}\right)\cdot \log\left(1-\sigma (p_{i})\right)\right)$$

where *N* denotes the total number of samples, σ(·) denotes a sigmoid function, and $p_{i}$ represents the probability that the ith sample is positive.

### 2.6. Evaluation Metrics

To enable a more objective and comprehensive comparison between the proposed model and previous studies, we select the same evaluation metrics used in prior research. These include Balanced Accuracy (BACC), Sensitivity (SN), Specificity (SP), Matthews Correlation Coefficient (MCC), and Area Under the ROC Curve (AUC). The AUC is particularly valuable, as it is independent of specific threshold settings, providing a more robust assessment of the model’s generalization performance. Balanced Accuracy (BACC) is also an important metric, as it accounts for both positive and negative samples, thereby mitigating potential bias caused by class imbalance. The formulas for calculating these metrics are as follows:

(12) $$BACC=\frac{SN+SP}{2}$$

(13) $$SN=\frac{TP}{TP+FN}$$

(14) $$SP=\frac{TN}{TP+FN}$$

(15) $$MCC=\frac{TP\times TN-FP\times FN}{\sqrt{\left(TP\times FN)(TP\times FP)(TN\times FP)(TN\times FN\right)}}$$

(16) AUC:AreaundertheROCCurve

In this context, TP and FP denote true positives and false positives, respectively. True positives refer to the number of samples that are correctly predicted as positive, while false positives represent the samples incorrectly predicted as positive. Similarly, TN and FN represent true negatives and false negatives. True negatives correspond to the samples correctly predicted as negative, whereas false negatives refer to the samples incorrectly predicted as negative.

### 2.7. Model Implementation

The model is constructed using PyTorch and its Geometric framework [31]. ADAM is employed as the optimizer, with a training batch size of 256. To address the class imbalance between positive and negative samples, the weights for positive and negative samples are set at a ratio of 10:1. The initial learning rate is set to 0.001, and it is reduced by 5% every 5 epochs. The model is trained for a total of 40 epochs. For hyperparameter optimization, a grid search strategy is employed, with the best combinations selected based on the AUC from the validation set.

## 3. Results

### 3.1. Pre-Trained Model Selection Experiment

**Figure 4:**
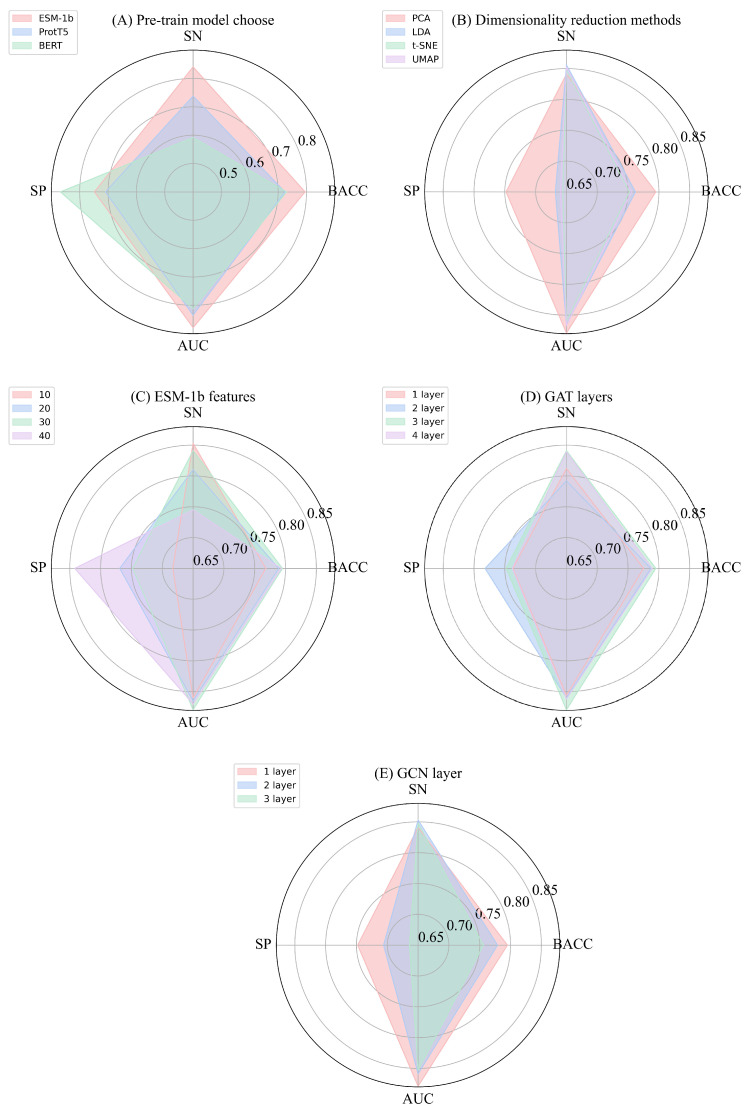
Five-fold cross-validation was performed for the experiments, covering the following aspects: (**A**) selection of the pre-trained model, (**B**) selection of dimensionality reduction methods, (**C**) selection of dimensions for ESM-1b features, (**D**) selection of GAT layers, and (**E**) selection of GCN layers.

In the process of selecting the pre-trained models, we conducted a five-fold cross-validation and compared the performance of BERT [32], ProtT5 [33], and ESM-1b models. Since these models extract feature vectors of different dimensions, we performed dimensionality reduction on the features before conducting the comparative experiments. As shown in Figure 4A, the ESM-1b model outperformed the others in terms of evaluation metrics such as AUC, BACC, and SN. This can be attributed to the fact that, although BERT and ProtT5 excel in capturing long-range dependencies and contextual information, the relatively short sequences in our study may limit their performance. In contrast, ESM-1b is capable of capturing the position and relative importance of amino acids in three-dimensional structures by considering their deep features. This ability not only enhances our understanding of how amino acids interact with each other but also provides insights into their functional roles within peptide segments. The deep features enable the model to identify the importance of key amino acid positions for biological activity, thereby improving prediction accuracy. After carefully considering both the experimental results and the model characteristics, we ultimately selected ESM-1b as the pre-trained model for our study.

### 3.2. Threshold Analysis for Classification

**Figure 5:**
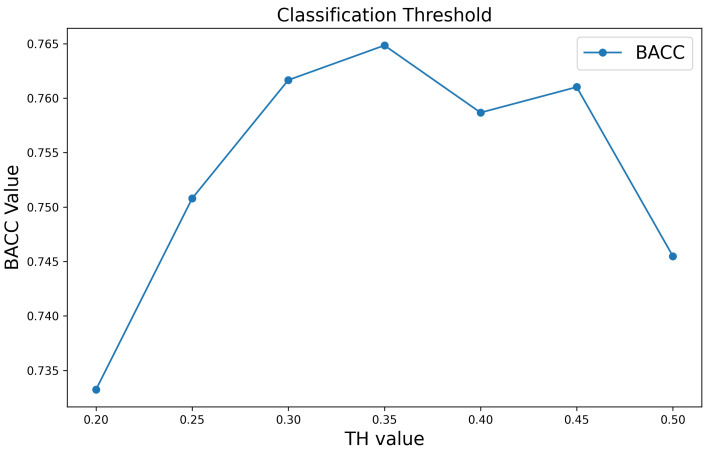
Comparison of the model’s performance across various classification thresholds using five-fold cross-validation.

Due to the substantial imbalance between positive and negative samples in the dataset, with a ratio of 0.122, selecting an appropriate classification threshold was crucial. To determine the optimal threshold, BACC was used as the evaluation metric, and five-fold cross-validation was performed on the training set. The results, as shown in Figure 5, indicate that setting the classification threshold to 0.35 yields the highest BACC, with the model performing best when it comes to distinguishing between negative and positive samples. Specifically, if the predicted probability of a positive sample, obtained from the softmax function, exceeds 0.35, the sample is classified as positive; otherwise, it is classified as negative. Thus, the classification threshold was set to 0.35 in this study.

### 3.3. Dimension Selection of ESM-1b Features

To effectively integrate the features obtained from ESM-1b with the other two feature types and ensure optimal prediction performance, it is essential to reduce the dimensionality of the 1280-dimensional ESM-1b features to a smaller number of dimensions [34]. In this study, several dimensionality reduction methods were compared, including linear discriminant analysis (LDA) [35], t-distributed stochastic neighbor embedding (t-SNE) [36], and uniform manifold approximation and projection (UMAP) [37]. These methods were evaluated by reducing the dimensionality of the ESM-1b features to the same dimension, as shown in Figure 4B. After comparison, PCA was chosen as the dimensionality reduction method. PCA effectively projects high-dimensional data into a lower-dimensional space while preserving as much variance as possible, thereby maintaining the primary structure of the data and reducing the number of features [26]. To ensure consistency and comparability across models, the same dimensionality reduction method was applied to all models. This approach allows for fair evaluation of performance differences between models. To determine the optimal dimensionality, experiments were conducted on the training set, testing four dimensions in intervals of 10, ranging from 10 to 40, with AUC as the evaluation metric. The dimension corresponding to the best result was selected for further analysis. Figure 4C presents the average results of five-fold cross-validation for each experiment. The results show that the best and most stable performance is achieved when the ESM-1b features are reduced to 30 dimensions. At this dimension, the difference between the ESM-1b features and those extracted from position encoding and one-hot encoding becomes minimal, and the reduction in dimensionality has little impact on the model’s performance. This helps retain the essential feature information, leading to the best results.

### 3.4. Analysis of GAT and GCN Layers

#### 3.4.1. Experiments on GAT Layers Selection

Using a five-fold cross-validation approach on the training set, we systematically evaluated various numbers of GAT layers to determine the optimal configuration. We tested different combinations of GAT layers, ranging from one to four, to examine how the number of layers affects model performance. As shown in Figure 4D, the three-layer GAT demonstrated significant improvements, with increases of 0.2% to 4.9% in SN and 1.7% to 2.3% in AUC, indicating that the three-layer GAT outperformed others in predicting IL-6-inducing peptides. The superior performance of the three-layer GAT may be attributed to its ability to capture and represent the complex structure and relationships between nodes in the graph more effectively by increasing the network depth and leveraging the flexibility of the attention mechanism. Although a four-layer GAT could theoretically offer greater model capacity, the limited volume of data in this study suggests that a four-layer GAT might overcomplicate the model, increase training difficulty, and potentially lead to overfitting. Considering both the model’s performance and training feasibility, we ultimately selected the three-layer GAT as a key module in the dual-channel architecture. This choice aims to balance predictive power with training efficiency, ensuring that the model captures the essential data characteristics while avoiding unnecessary complexity.

#### 3.4.2. Experiments on GCN Layer Selection

By evaluating the results of five-fold cross-validation on the training set, we determined the optimal number of GCN layers. One-layer, two-layer, and three-layer GCNs were tested. As shown in Figure 4E, the one-layer GCN outperformed the others. Specifically, compared to the two-layer GCN, the one-layer GCN improved the BACC on the training set by 1.6% to 3.9%, the MCC by 1.6%, and the AUC by 2% to 2.6%. Given the relatively short sequence length and simple graph structure in our dataset, a one-layer GCN was sufficient to capture valuable information from the graph. Increasing the number of layers did not yield further performance gains and may have unnecessarily increased the model’s complexity. Therefore, considering both performance and model simplicity, we chose to use a one-layer GCN as the second channel in the dual-channel feature extraction module. This decision ensured that the model maintained efficient performance while preserving structural simplicity.

#### 3.4.3. Additional Parameter Selection Experiments

In this section, we discuss the impact of model parameters on the performance of DGIL-6, specifically focusing on the amino acid contact threshold, the number of GAT attention heads, and the embedding dimension of hidden layers. These parameters were selected based on AUC and SN from the results of five-fold cross-validation.

**Figure 6:**
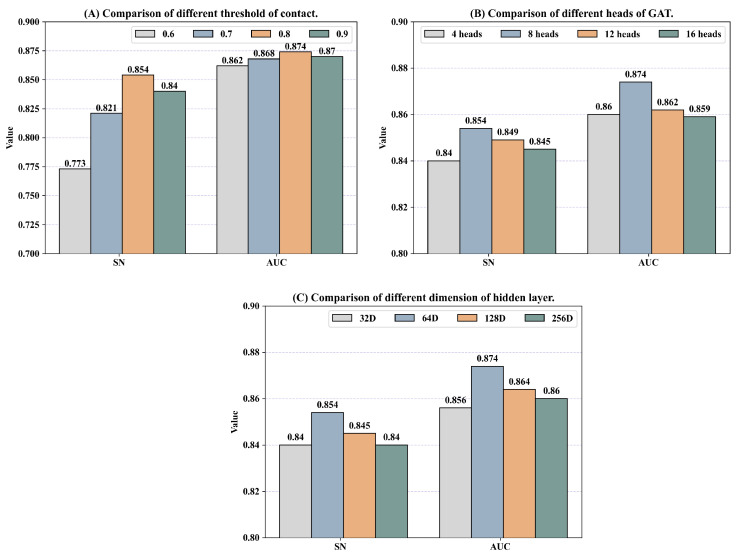
Five-fold cross-validation was conducted in the experiments. (**A**) Selection of the amino acid contact threshold. (**B**) Selection of the number of GAT attention heads. (**C**) Selection of the hidden layer embedding dimension.

The selection of the amino acid contact threshold significantly influences the composition stage. Firstly, we tested thresholds of 0.6, 0.7, 0.8, and 0.9. As shown in Figure 6A, a lower threshold generates excessive edge information, negatively impacting the model’s performance, while a higher threshold may exclude key edge information. Therefore, we selected a contact threshold of 0.8. Next, the multi-head attention mechanism in the GAT layer was evaluated. We tested different numbers of attention heads (4, 8, 12, and 16). As shown in Figure 6B, DGIL-6 performed better as the number of attention heads increased, but its performance declined when the number exceeded eight. Consequently, we chose eight attention heads for the GAT layer. Finally, we examined the impact of the embedding dimension of hidden layers on model performance. Values of 32, 64, 128, and 256 were tested. As shown in Figure 6C, DGIL-6 achieved optimal performance with an embedding dimension of 64.

### 3.5. Ablation Experiments

#### 3.5.1. Ablation Experiments of Node Features

To evaluate the impact of different node characteristics on model performance, we conducted a series of ablation experiments on the testing set, with the results presented in Table 2. The experimental results demonstrate that combining ESM-1b-extracted features with position encoding and one-hot encoding significantly improved the model’s predictive performance, particularly in BACC and AUC metrics. This feature fusion strategy enhanced the model’s representation capability: one-hot encoding supplied categorical information about nodes, position encoding facilitated the capture of amino acid positions within peptide sequences, and ESM-1b features enabled the model to capture biological signals related to protein functionality. Notably, the ESM-1b-extracted features significantly enhanced the model’s performance, with AUC increasing by 5%, MCC by 10.5%, BACC by 6.7%, and SN by 8.2%. The relative positions of amino acids in peptide sequences directly influenced their functions and interactions. However, graph neural networks often struggle to capture sequential positions when processing sequence data. The introduction of position encoding compensates for this limitation. By providing precise positional information for each node, position encoding enabled the model to better interpret node positions within peptide sequences [38]. The experimental results show that the AUC increased by 2.5%, MCC by 1.1%, the SN increased by 9.6%, and the BACC increased by 2.1%. Additionally, while one-hot encoding is less effective than ESM-1b features, the amino acid composition information it provides remains vital to the model’s performance. It resulted in the AUC increasing by 3.1%, MCC increasing by 4.5%, SP increasing by 3.4%, SN increasing by 1.6%, and BACC increasing by 2.4%. One-hot encoding allows the model to effectively capture the diversity of amino acids and their influence on protein functionality, thereby driving these improvements. These findings underscore the distinct contributions and synergistic effects of various features in enhancing the model’s predictive capability.

#### 3.5.2. Ablation Experiments of Dual-Channel Feature Extraction

Ablation experiments were conducted on the testing set to evaluate the effectiveness of the dual-channel feature extraction module and determine which component had the greatest impact on the performance of the model. As presented in Table 3, “No-GAT” refers to the model with the GAT module removed, while “No-GCN” represents the model with the GCN module excluded.

When the GAT module was removed from the model, a 6.1% decrease in AUC, a substantial 15% decrease in MCC, a 3.3% decrease in BACC, and a 15.4% decrease in SP were observed. Interestingly, SN increased by 1.3%, indicating that while GAT excels at predicting negative samples, it is relatively less effective at identifying positive samples. In contrast, removing the GCN module led to a 2.6% decrease in AUC, a 2.8% decrease in MCC, a 1.7% decrease in BACC, an 8.2% decrease in SN, and a 5% increase in SP. These results suggest that GCNs are more effective when it comes to predicting positive samples. The observed differences may stem from the unique characteristics of the two channels: GCN leverages local neighborhood information to learn node features, whereas GAT uses an attention mechanism to assign varying importance weights to neighbors, capturing more nuanced relationships between nodes. By integrating these two mechanisms, the model can comprehensively capture both global and local features within the graph, enhancing its overall predictive performance. These findings highlight the complementary roles of GAT and GCN modules and their critical contributions to improving the prediction accuracy of IL-6-inducing peptides.

### 3.6. Comparison Experiments with Existing Methods

To compare the proposed model against existing prediction tools, we conducted comparative experiments on the testing set. The evaluated IL-6-inducing peptide prediction models include IL-6Pred, StackIL6, and MVIL6. Performance metrics of IL-6Pred, StackIL6, and MVIL6 were derived from prior studies, with references specifically citing the MVIL6 study. The comparative results of these methods are summarized in Table 4.

As shown in Table 4, DGIL-6 showed improvements across several key performance metrics: AUC increased by 1.9% to 7.2%, BACC increased by 0.4% to 9.5%, MCC increased by 0.6%to 7.8%, SP increased by 0.5% to 7.2%, and SN increased significantly, by 1.3% to 12.3%. These advancements can be largely attributed to the utilization of the pre-trained ESM-1b model for extracting amino acid features, which enriches the model with more comprehensive biological information, thereby enhancing prediction accuracy and reliability. The DGIL-6 achieves complementary features by integrating 3D structure-based graph representations with amino acid-based depth features, resulting in superior performance in IL-6-inducing peptide prediction.

### 3.7. Hierarchical Clustering Analysis of Dual-Channel Features

**Figure 7:**
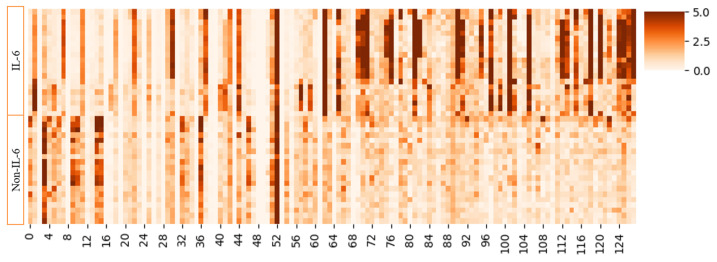
The feature visualization results of two channels presented via hierarchical clustering on positive and negative samples. The vertical axis represents different individual samples. The horizontal axis indicates the feature indices, with features 0–63 derived from the GAT channel and features 64–127 derived from the GCN channel. The heatmaps utilize color gradients to visualize normalized feature values, where darker colors indicate higher values and lighter colors indicate lower values.

To visually demonstrate the features extracted by different channels of the DGIL-6 model, we conducted a hierarchical clustering analysis after concatenating the features from the GAT and GCN channels. Hierarchical clustering was selected because it effectively groups similar features into a hierarchical structure, facilitating a clear observation of feature similarity. This method is particularly well suited for exploring the intrinsic structure of data and identifying feature patterns. The visualization helps to quickly pinpoint similar features and provides insight into their distribution and significance across the entire dataset. The results of the hierarchical clustering analysis [39] are presented in Figure 7. As shown in the characteristic distribution of IL-6-inducing peptides and non-IL-6-inducing peptides in Figure 7, regions with larger eigenvalues tend to form clusters. This finding suggests that features derived from the model are similar for sequences with similar functions but significantly different for sequences with distinct functions. It is noted that larger eigenvalues indicate that these features play a more significant role in distinguishing different categories.

From the feature distributions extracted by GAT and GCN in Figure 7, it can be observed that the two channels capture distinct features of the data. GAT focuses on neighboring nodes that exert the greatest impact on classification, enhancing the model’s sensitivity to critical features. In contrast, GCN effectively smooths node features, bringing similar nodes closer in feature space, making it well suited for capturing overall graph structural information. This complementary understanding enables the model to better identify structural patterns in peptides. These distinct contributions improve the classification of IL-6-inducing peptides, further validating the effectiveness of the dual-channel feature extraction module. The clustering results in Figure 7 demonstrate that the extracted features effectively separate IL-6-inducing peptides from non-IL-6-inducing peptides, highlighting their strong discriminative capacity. However, some features exhibit low values in both IL-6 inducing and non-IL-6-inducing peptides, suggesting these features may be shared between the two groups, making it difficult for the model to distinguish them. Further optimization of these features could enhance the model’s performance and reduce false positives.

## 4. Conclusions

We developed a prediction tool, DGIL-6, for identifying IL-6-inducing peptides using graph neural networks and predicted structural information. DGIL-6 represents each peptide sequence as a graph, where amino acids serve as nodes, and the adjacency matrix, representing relationships between nodes, is computed based on the predicted residue contact graph of the peptide sequence. The graph’s node features are composed of one-hot encoding, positional encoding, and features extracted using ESM-1b. To extract multi-view features, a dual-channel feature extraction module was implemented, combining a three-layer multi-head GAT with a one-layer GCN. The effectiveness of the proposed method was validated through various experiments, including comparison studies, ablation analysis, and feature visualizations. Although we achieved promising results, there is still room for improvement. Future work could address the challenge of insufficient positive samples using transfer learning or enhance graph construction performance by leveraging structural information of protein sequences obtained from advanced large-scale models.

## Figures and Tables

**Table 1:** An overview of the dataset.

Dataset	Positive Data	Negative Data	Ratio
Training set	292	2393	0.122
Test set	73	598	

**Table 2:** Ablation experiment results of node features.

Node Features	BACC	SN	SP	MCC	AUC
No-One-hot	0.814	0.863	0.765	0.426	0.871
No-Position	0.817	0.780	**0.854**	0.460	0.877
No-ESM-1b	0.771	0.794	0.749	0.366	0.852
DGIL-6	**0.838**	**0.876**	0.799	**0.471**	**0.902**

Note: The best value for each indicator is shown in bold.

**Table 3:** Ablation experiment results of dual-channel feature extraction.

Dual-Channel	BACC	SN	SP	MCC	AUC
No-GAT	0.805	0.863	0.645	0.321	0.841
No-GCN	0.821	0.794	**0.849**	0.453	0.876
DGIL-6	**0.838**	**0.876**	0.799	**0.471**	**0.902**

Note: The best value for each indicator is shown in bold.

**Table 4:** Comparative experimental results with existing methods.

Tool	BACC	SN	SP	MCC	AUC
IL-6Pred	0.743	0.753	0.732	-	0.830
StackIL6	0.795	0.849	0.741	0.393	0.841
MVIL6	0.834	0.863	**0.804**	0.465	0.883
DGIL-6	**0.838**	**0.876**	0.799	**0.471**	**0.902**

Note: The best value for each indicator is shown in bold; "-" represents a null value.

## Data Availability

The source code and dataset used in this study are available at https://github.com/IL-6Li/DGIL-6 (accessed on 7 January 2025).
